# Anticancer-Drug-Related Cardiotoxicity from Adjuvant Goserelin and Tamoxifen Therapy

**DOI:** 10.3390/jcm14020484

**Published:** 2025-01-14

**Authors:** Olivia Manfrini, Edina Cenko, Maria Bergami, Jinsung Yoon, Jelena Kostadinovic, Darko Zdravkovic, Marija Zdravkovic, Raffaele Bugiardini

**Affiliations:** 1 Laboratory of Epidemiological and Clinical Cardiology, Department of Medical and Surgical Sciences, University of Bologna, 40138 Bologna, Italy; olivia.manfrini@unibo.it (O.M.); edina.cenko2@unibo.it (E.C.); maria.bergami@unibo.it (M.B.); 2 IRCCS Azienda Ospedaliero-Universitaria di Bologna, Sant’Orsola Hospital, 40138 Bologna, Italy; 3 Google Cloud AI, Sunnyvale, CA 94043, USA; jsyoon0823@gmail.com; 4 Department of Oncology, University Hospital Medical Center, Bezanijska Kosa, 11000 Belgrade, Serbia; jkostadinovicns@gmail.com; 5 Department of Surgical Oncology, University Hospital Medical Center, Bezanijska Kosa, 11000 Belgrade, Serbia; drdarkozdravkovic@gmail.com; 6 Faculty of Medicine, University of Belgrade, 11000 Belgrade, Serbia; sekcija.kardioloska@gmail.com; 7 Department of Cardiology, University Hospital Medical Center, Bezanijska Kosa, 11000 Belgrade, Serbia

**Keywords:** cardiotoxicity, breast cancer, left ventricular dysfunction, endocrine therapy, goserelin, tamoxifen

## Abstract

**Background:** Breast cancer is a prevalent malignancy with rising incidence globally. Advances in endocrine therapy have improved outcomes for premenopausal women with hormone receptor-positive breast cancer. However, these treatments may induce menopause-like states, potentially elevating cardiovascular risks, including left ventricular (LV) dysfunction. This study aims to evaluate the impact of one year of adjuvant endocrine therapy with goserelin and tamoxifen on LV function in premenopausal breast cancer patients. **Methods:** The ISACS cardiovascular toxicity (NCT01218776) is a pilot multicenter registry of breast cancer patients referred to hospitals for routine surveillance, suspected, or confirmed anticancer-drug-related cardiotoxicity (ADRC). Patients may be enrolled retrospectively (1 year) and prospectively. The pilot phase focused on the available data on combined goserelin and tamoxifen therapy for breast cancer and its impact on LV disfunction at 1-year follow-up. Inverse probability of treatment weighting (IPTW) analysis of the ISACS registry was performed assigning 70 patients to combined endocrine therapy (goserelin and tamoxifen). Controls consisted of 120 patients with no adjuvant combined goserelin and tamoxifen therapy. None of the patients developed distant metastasis. Primary outcome measures were as follows: low LV function in women as defined by a left ventricular ejection fraction (LVEF) < 65% and subclinical LV dysfunction as defined by a 10-percentage point decrease in LVEF. **Results:** In the overall population, combined goserelin and tamoxifen therapy did not affect the mean LV function compared with controls at 3-, 6-, and 12-month follow-up (65.7 ± 2.7% versus 65.3 ± 2.1%, *p* value = 0.27; 65.5 ± 2.9% versus 65.1 ± 2.5%, *p* value = 0.34; 65.0 ± 3.2% versus 64.6 ± 3.1%, *p* value = 0.29, respectively). The mean LVEF reduction in patients who did or did not receive combination therapy for 12 months was small and approximately similar (1.03 ± 2.5% versus 1.16 ± 2.9%, *p* value = 0.73). Using IPTW analyses, there were no significant associations between combined therapy and low LV function (risk ratio [RR]: 1.75; 95% CI: 0.71–4.31) or subclinical LV dysfunction (RR: 1.50; 95% CI: 0.35–6.53) compared with controls. **Conclusions:** One year of endocrine therapy with goserelin and tamoxifen does not cause ADRC in patients with invasive breast cancer. Findings are independent of the severity of the disease. Results may not be definitive without replication in studies with larger sample size.

## 1. Introduction

Breast cancer is a common malignancy with rising incidence worldwide [1,2]. Advances in chemotherapy and endocrine therapy, including ovarian function suppression via gonadotropin-releasing hormone (GnRH) analogues, selective estrogen receptor modulators, and aromatase inhibitors, have significantly improved prognosis [3]. For premenopausal women, GnRH analogues suppress ovarian function, reduce estrogen production, and slow cancer progression [3]. Aside from standard chemotherapy agents, for early hormone receptor (HR)-positive breast cancer, current guidelines recommend endocrine therapy in the form of GnRH analogues, which may be combined with tamoxifen, a selective estrogen receptor modulator [3].

While endocrine therapy effectively reduces the risk of breast cancer recurrence and metastasis, its long-term cardiovascular implications warrant closer scrutiny. GnRH analogues, commonly used in premenopausal women, may increase cardiovascular risk by inducing a menopause-like state [2]. This state adversely affects lipid metabolism, glucose regulation, and vascular health, potentially elevating the risk of ischemic heart disease [2]. Specifically, these therapies can impair endothelial function, destabilize atherosclerotic plaques, and disrupt vasomotor regulation [4,5,6,7,8]. These changes may contribute to clinical and subclinical ischemic heart disease, which, in turn, can impair left ventricular (LV) function. The cardiovascular risks are further compounded in patients receiving concomitant chemotherapy, especially if anthracycline-based [9,10,11,12]. Similarly to alkylating agents like cyclophosphamide and platinum compounds, anthracyclines are well documented for their cardiotoxic effects, including LV dysfunction [9,10,11,12,13,14], which may exacerbate the cardiovascular burden associated with GnRH analogue-induced menopause. On the other hand, the potential detrimental effects of GnRH analogues, either alone or in combination with anthracyclines, may be mitigated by the concurrent administration of tamoxifen. Tamoxifen has been shown to reduce the risk of ischemic heart disease, even when used for up to 10 years [15]. However, further evidence is needed to confirm or refute this hypothesis. Consequently, the long-term cardiac safety of breast cancer treatments, particularly in estrogen receptor (ER)-positive cases requiring extended treatment durations, remains an area of active investigation [16].

The current study explores the impact of endocrine therapy on LV function in premenopausal breast cancer patients. Specifically, it evaluates the risk of LV dysfunction in patients treated with a combination of goserelin, a synthetic analogue of luteinizing hormone-releasing hormone, and tamoxifen for one year, compared with those who did not receive adjuvant endocrine therapy or who received solely tamoxifen. The study also investigates treatment-specific endocrine side effects based on immunohistochemical stratification, with a focus on human epidermal growth factor receptor 2 (HER2) expression as the optimal treatment strategy for HER2-positive tumours, particularly in the context of endocrine therapy, is not fully elucidated.

## 2. Methods

### 2.1. Patients

**Figure 1:**
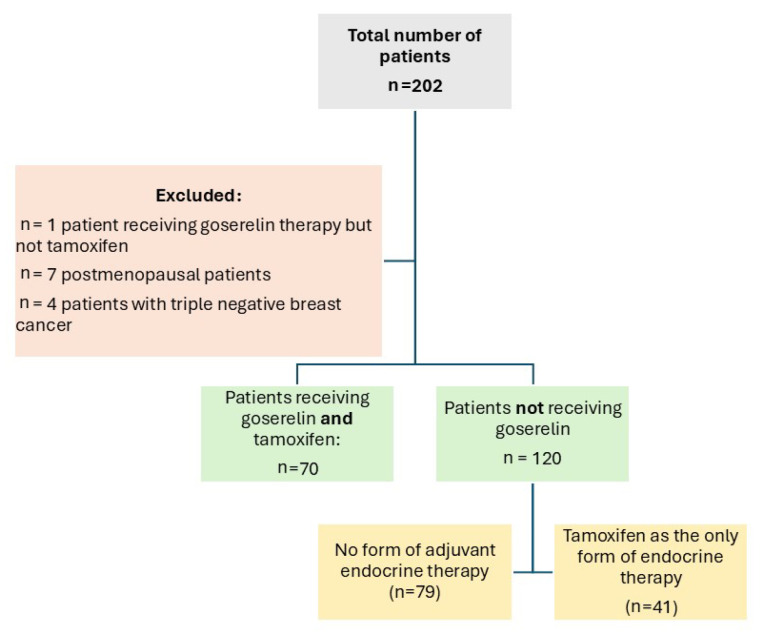
Study flow chart.

From 2016 to 2018, the ISACS cardiovascular toxicity (NCT01218776) registry enrolled, in a pilot phase, 70 premenopausal women diagnosed with invasive operable breast cancer who were given combined therapy with goserelin (3.6 mg subcutaneously once every 28 days) and tamoxifen (40 mg orally once daily). The control population consisted of patients with no form of adjuvant endocrine therapy (n = 79) or patients who received only tamoxifen but no goserelin (n = 41) (Figure 1). Both groups could receive concurrent standard adjuvant chemotherapy (including anthracyclines, trastuzumab, paclitaxel, and docetaxel) and locoregional radiotherapy. As current guidelines indicate, premenopausal patients affected by hormone-sensitive malignancies as the optimal target for endocrine treatment with GnRH analogues [17], postmenopausal women (n = 7 patients), as well as those diagnosed with triple negative breast cancer (n = 4) were excluded from the analysis. A single patient treated with goserelin but not tamoxifen was also excluded to avoid confounding. None of the patients developed distant metastasis. All patients were followed-up and screened for ADRC with electrocardiography and transthoracic echocardiography at baseline (prior to any cancer-related treatments taking place) and at 3 months, 6 months, and 1 year after chemotherapy and radiotherapy.

The study was approved by the local research ethics committee. Because patient information was collected anonymously, the institutional review board waived the need for individual informed consent. The study adheres to the principles of the Declaration of Helsinki.

### 2.2. Tumour Size, Lymph Node Tumour Grading and Hormone Receptor Status

Tumour size (T) was categorized into three groups according to clinical guidelines: stage T1 (≤20 mm), stage T2 (21–50 mm), and stage T3 (>50 mm). Lymph node involvement was defined as N0 (there is no spread to nearby lymph nodes), N1 (cancer has spread to fewer than 3 lymph nodes located on the underarm or has spread to any number of lymph nodes located near the breastbone-internal mammary lymph nodes), and N2 (cancer has spread to between 4 and 9 lymph nodes of the underarm or has enlarged the lymph nodes of the internal mammary area). Tissue samples were processed according to routine procedures. Hormone receptor staining (ER, progesterone receptor (PR), and HER2) was assessed retrospectively with immunohistochemistry using current standard methodology.

### 2.3. Outcome Measure

The primary outcome was the relationship between measures of left ventricular ejection fraction (LVEF) at baseline, and those during follow-up at 3, 6 and 12 months. Low LV function in women was defined as an LVEF < 65% [18]. Subclinical LV dysfunction was defined as a 10-percentage point decrease in LVEF to a value < 53% [19]. According to the ESC, LVEF assessment can be performed by echocardiography, cardiac nuclear imaging, and cardiac magnetic resonance (CMR) [20,21].

### 2.4. Statistical Analyses

Patient characteristics were stratified according to treatment group: patients with combined goserelin and tamoxifen therapy versus those with no combined therapy. Baseline characteristics were reported as percentages for categorical variables and means with standard deviation (SD) for continuous variables (Table 1, Table 2 and Table 3). We had complete data on outcomes and covariates. Estimates of odds ratios (ORs) or risk ratios (RRs) and associated 95% CIs were obtained using logistic regression or inverse probability of treatment weighting models, respectively. We used logistic models to evaluate the effect of goserelin and tamoxifen therapy on LV function. Three models were run that incrementally added covariates. The first model included only age. Model 2 then additionally adjusted for the use of radiotherapy. Model 3 included additional therapeutic factors that have been suggested as potential reasons for variation in LV function, specifically use of adjuvant medical therapies (anthracycline, trastuzumab, paclitaxel and docetaxel). By adjusting for patient characteristics and main therapeutic factors incrementally, we attempted to understand the contribution of concurrent treatment to outcomes. Inverse probability weighting was calculated using the propensity score to create a sample in which the distribution of measured baseline covariates was independent from the use of combined goserelin and tamoxifen therapy [22]. Because of the instability that can be induced by extreme weights, stabilized weights were used that also preserve the original sample size. We created a threshold for weights to avoid the impact of the outliers. We used 0.01 as the threshold of the propensity weighting. As the sample size was too small, *p* values instead of standardized differences after weighting were calculated to ensure balanced treatment groups with respect to baseline characteristics. Groups were considered balanced when the *p* value was <0.05 [23]. To account for differences in patient-level characteristics and illness severity among groups, we prespecified the following covariates for inclusion in the models: demographics, cardiovascular risk factors, tumour size and lymph node tumour grading, and in-hospital treatment (mastectomy and adjuvant medical therapies: anthracycline, trastuzumab, paclitaxel, and docetaxel). A *p* value < 0.05 was taken to indicate that the difference between the effects in goserelin and tamoxifen recipients versus nonrecipients was unlikely to have occurred simply by chance. All statistical analyses were performed using R version 3.4.4.

### 2.5. Inverse Probability of Treatment Weighting Analyses

We used Inverse Propensity-Score Weighting to balance the distribution of covariates between two patient groups. Note that we use logistic regression to estimate the propensity scores ({P}(Z = 1|x)). If *e* denotes the estimated propensity score (i.e., e = \hat{P}(Z = 1|x), where the patient x is included in patient group 1, then 1 − e = \hat{P}(Z = 0|x)), and then, the original sample is weighted by the following weights: Z/e + (1 − Z)/1 − e where Z represents the patient group. For instance, endocrine therapy group (Z = 1) is assigned a weight equal to the reciprocal of the propensity score (1/e), while controls (Z = 0) are assigned a weight equal to the reciprocal of one minus the propensity score (1/1 − e). The weighting procedure for each sample balances the covariate distributions between two patient groups.

## 3. Results

Patient and tumour characteristics of the 190 HR-positive individuals are shown in Table 1. Of these patients, 70 underwent combination of goserelin and tamoxifen. The control population consisted of patients with no adjuvant endocrine therapy (n = 79) or patients who underwent only tamoxifen (n = 41). The mean age was 37.8 ± 4.8 years for patients with combined therapy and 46.0 ± 9.1 for controls (*p* value < 0.001). The majority of patients were PR-positive (100% in patients with combined therapy and 83.3% in controls, *p* value < 0.001). One third of patients were HER2-positive (28.6% in patients with combined therapy and 30.8% in controls: *p* value = 0.74). According to guideline protocols, lymph node-positive patients (n = 73) received neoadjuvant chemotherapy. Oncology management is shown in Table 2.

In the overall population, combined goserelin and tamoxifen therapy did not affect the mean LV function compared with controls at 3-, 6-, and 12-month follow-up (65.7 ± 2.7% versus 65.3 ± 2.1%, *p* value = 0.27; 65.5 ± 2.9% versus 65.1 ± 2.5%, *p* value = 0.34; 65.0 ± 3.2% versus 64.6 ± 3.1%, *p* value = 0.29, respectively). The mean LVEF reduction in patients who did or did not receive combination therapy for 12 months was small and approximately similar (1.03 ± 2.5% versus 1.16 ± 2.9%, *p* value = 0.73) (Table 3).

### 3.1. The Effects on LV Function Assessed by Multivariable Logistic Regression Analysis

Multivariable analyses assessed the effect on LF function in patients treated with or without goserelin and tamoxifen therapy (Table 4). Outcome variables were low LV sex-specific function (LVEF < 65%) and subclinical LV dysfunction (10-percentage point decrease in LVEF). We used as a reference level for comparison the outcomes of patients without endocrine therapy. As such, the predicted model included a beta coefficient and the corresponding OR for “patients treated with goserelin and tamoxifen therapy” and a beta coefficient and the corresponding OR for “patients treated with tamoxifen therapy alone”. Three models were run that incrementally added covariates. The first model included only age. Model 2 then additionally adjusted for the use of radiotherapy. Model 3 included additional therapeutic factors that have been suggested as potential reasons for variation in LV function, specifically use of adjuvant medical therapies (anthracycline, trastuzumab, paclitaxel and docetaxel). In Model 1, no significant adverse effects from the combination of goserelin and tamoxifen were seen compared with control therapy.

These associations did not differ according to treatment with radiotherapy or adjuvant medical therapies (model 2 and 3). The change in the ORs we observed in our analysis on combined goserelin and tamoxifen therapy versus control therapy, from an OR of 0.95 when adjusting only for age to an OR of 1.24 after additionally adjusting for adjuvant therapies and radiotherapy, is a hypothesis-generating finding. This shift suggests a slight increase in the odds of LV dysfunction with such treatments, but the confidence interval is broad, indicating no clear statistical significance.

### 3.2. Balancing Covariates and Outcomes

The characteristics of patients receiving goserelin and tamoxifen therapy and those of the propensity-score weighted controls were well balanced with *p* values < 0.05 (Table 5).

**Figure 2:**
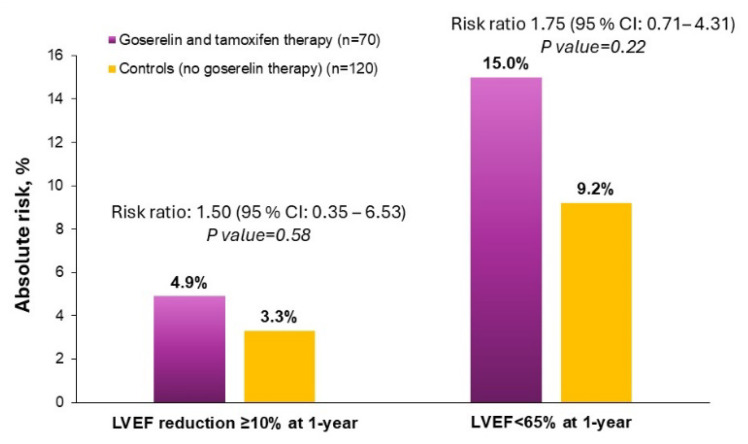
Risk ratios of goserelin and tamoxifen therapy to controls for primary outcomes of subclinical LV dysfunction and low LV function.

Compared with control therapy, combined goserelin and tamoxifen use was not significantly associated with a significantly increased risk of low LV function (RR: 1.75; 95% CI: 0.71–4.31) and subclinical LV dysfunction (RR: 1.50; 95% CI: 0.35–6.53) (Figure 2).

## 4. Discussion

The current investigation of premenopausal patients with ER-positive breast cancer demonstrates that there was no significant reduction in the functional parameters of cardiac performance of one year of combination of goserelin and tamoxifen use compared with tamoxifen use alone or no adjuvant endocrine therapy.

Today, the addition of ovarian function suppression by goserelin to tamoxifen is recommended to clinical high-risk patients, often defined by lymph node involvement, high grade, high proliferation, high genomic risk scores, or an age below 40 years [24]. How goserelin and tamoxifen work on a mechanistic level, alone or in combination, is not fully understood. In brief, goserelin increases GnRH in the pituitary gland resulting in postmenopausal levels of circulating estrogens [25]. Tamoxifen is a selective estrogen receptor modulator. Its mechanism of action is unique to its dual role as both an agonist and an antagonist of the ER. Some studies argue that adding goserelin to tamoxifen may more effectively reduce estrogen levels compared to tamoxifen alone. Others speculate that the agonistic effects of tamoxifen may counteract the estrogen-depleting effects of goserelin [26].

Despite the potential benefits in terms of disease progression and overall survival from breast cancer, the use of GnRH analogues may not be risk-free in the long run. In male patients treated for prostate cancer, GnRH analogues have been demonstrated to increase the risk of coronary heart disease (HR, 1.16; *p* value < 0.001), myocardial infarction (HR, 1.11; *p* value = 0.03), and sudden cardiac death (HR, 1.16; *p* value = 0.004) [27]. Unfortunately, the same level of evidence is not available for women, with a very limited number of studies having investigated the direct role of this drug class in influencing development of IHD and cardiac dysfunction. Prior studies have suggested that use of GnRH analogues may be associated with a reduced risk of IHD, but the results may have been hindered by the long enrollment period (from 2000 to 2018) [28]. On the other hand, indirect evidence, including animal studies, suggests that GnRH analogues may increase the risk of cardiovascular risk factors like diabetes and could thus manifest their detrimental effects on cardiovascular health later in life [29]. However, such hypotheses are, to our knowledge, yet to be verified.

In the current study, the main side effects were reasonable. Severe side effects on LV function (LVEF < 50%) were not observed. This indicates that goserelin, in combination with tamoxifen, is safe. Our results also showed that combined goserelin and tamoxifen therapy does not increase subclinical LV dysfunction compared with controls. Current vision accepts cardiotoxicity as a decline in LVEF of at least 10% to a final value of under 53% [19]. In our analysis, subclinical LV dysfunction occurred more frequently with one year of combined goserelin and tamoxifen exposure (4.9%) compared with one year of control therapy (3.3%). However, such differences were small and not significant. On the other hand, the width of the confidence interval and the size of the *p* value are related, and the width of a confidence interval decreases as the sample size increases. In summary, our sample size is too small to draw definitive conclusions on drug-related cardiotoxicity based on measurements of subclinical LV dysfunction.

In our study, we assessed as outcome measure the incidence of LVEF < 65% at 12 months, because prior work has shown that there are sex-related differences in normal LVEF values, with women generally having higher LVEF levels than men [18]. We hypothesized, therefore, that using a single, sex-neutral LVEF threshold for diagnosis of LV dysfunction may lead to under-diagnosis of drug-related cardiotoxicity in women. As such, we choose the LVEF cut-off values of normal (55–65%) and high (>65%) based on previously reported reference ranges [30]. There was an increase in the incidence of low (<65%) LVEF in the treatment group receiving a combination of goserelin and tamoxifen (15.0%) compared with the control group (9.2%). The relative risk was 1.75, suggesting that the treatment group had a 75% higher risk of developing low LV function compared with the control group. However, the wide confidence interval ranging from 0.71 to 4.31 indicates a significant degree of uncertainty in the risk estimate. While the increase in the risk of low LV function was not statistically significant, this finding raises new queries on the cardiac safety of combined goserelin and tamoxifen therapy. As such, it highlights the need for careful cardiac monitoring and, possibly, the development of sex-specific strategies to document this risk. Larger studies with longer follow-up would be needed to confirm these findings.

Our analysis was performed using IPTW. Inverse probability of treatment weighting has several advantages over other methods used to control for confounding, such as multivariable regression. IPTW can effectively balance covariates across treatment groups, reducing confounding biases. It is particularly useful when dealing with a large number of covariates. Unlike traditional regression models, IPTW does not require specifying the functional form of the relationship between covariates and the outcome. Finally, IPTW is beneficial in situations where outcomes are rare, as it does not suffer from the small number problem that can affect logistic regression. The use of IPTW is a critical issue in a setting where patients received various adjuvant therapies like anthracycline, trastuzumab, paclitaxel, and docetaxel. The concurrent use of these therapies introduces complexity in attributing outcomes, like LV dysfunction, to any single treatment. Understanding the isolated impact of goserelin and tamoxifen on LV function among these treatments is challenging but essential. This task is achieved by IPTW that mimics the conditions of a randomized controlled trial, attempting to isolate the effects of goserelin and tamoxifen from those of other treatments.

There are some limitations to this study. First, potential confounding and bias by intent to treat cannot be ruled out given the study’s observational nature. Inverse probability of treatment weighting has minimized such confounding. Second, all patients in our cohort are White, precluding any assessment of racial variations in response to goserelin and tamoxifen therapy. Third, the sample size is small, and, therefore, caution should be taken in the interpretation of the results. Fourth, assessment of LV function was based on LVEF as performed by routine echocardiography. Advanced echocardiography techniques, like strain imaging, can detect subtle changes in cardiac function even before a reduction in LVEF is evident. In addition, CMR can provide more detailed information about cardiac structure and function. Data on other laboratory parameters that could act as markers for cardiotoxicity (i.e., troponins, NT-proBNP) [31] were also not reported in the present registry. Finally, the timeframe of one-year follow-up may be too short to capture the long-term benefits of the therapy, particularly regarding recurrence prevention and survival. Patients with ER-positive breast cancer have a steady long-term risk of developing distant metastatic recurrences, with a large proportion of these events occurring beyond ten years after primary diagnosis [32]. Given this, longer follow-up is needed to understand the true endocrine treatment benefit in ER-positive breast cancer.

## 5. Conclusions

In conclusion, one year of combination of goserelin and tamoxifen therapy was not primarily associated with clinically significant LV dysfunction. Subclinical decreases in LVEF were seen in 4.9% of patients receiving combined goserelin and tamoxifen and 3.3% of those receiving control therapy. This observation, although not conclusive, highlights the need for careful cardiac monitoring of patients undergoing such treatments. Our findings also underscore the need for personalized medical approaches that consider sex-based physiological differences, ensuring that women receive appropriate evaluation and management for treatment-related cardiotoxicity. This includes reconsidering the use of uniform LVEF thresholds and potentially adopting sex-specific criteria for more accurate assessment and intervention.

## Figures and Tables

**Table 1:** Baseline characteristics stratified by concomitant use of adjuvant goserelin and tamoxifen.

Characteristics	Overall Population n = 190	Goserelin and Tamoxifen n = 70	No Goserelin n = 120	*p* Value
Age, mean ± SD, years	42.9 ± 8.7	37.8 ± 4.8	46.0 ± 9.1	<0.001
Premenopausal, n (%)	116 (61.1)	70 (100)	46 (38.3)	<0.001
Hypertension, n (%)	15 (7.9)	5 (7.1)	10 (8.3)	0.76
Hypercholesterolemia, n (%)	5 (2.6)	2 (2.9)	3 (2.5)	0.88
Diabetes, n (%)	10 (5.3)	7 (5.8)	3 (4.3)	0.64
Current smoking, n (%)	94 (49.5)	34 (48.6)	60 (50.0)	0.84
Obesity, n (%)	52 (27.4)	34 (28.3)	18 (25.7)	0.69
Prior hormone therapy, n (%)	21 (11.1)	7 (10.0)	14 (11.7)	0.72
Cancer characteristics				
Left breast, n (%)	113 (59.5)	33 (47.1)	80 (66.7)	0.008
IDC, n (%)	152 (80.0)	44 (62.9)	108 (90.0)	<0.001
ER-positive n (%)	150 (79.0)	70 (100.0)	80 (66.7)	<0.001
PR-positive, n (%)	170 (89.5)	70 (100.0)	100 (83.3)	<0.001
HER2-positive, n (%)	57 (30.0)	20 (28.6)	37 (30.8)	0.74
Stage T1, n (%)	20 (10.5)	13 (18.6)	7 (5.8)	0.006
Stage T2, n (%)	151 (79.5)	47 (67.1)	104 (86.7)	0.001
Stage T3, n (%)	19 (10.0)	10 (14.3)	9 (7.5)	0.13
N0, n (%)	117 (61.6)	27 (38.6)	90 (75.0)	<0.001
N1, n (%)	39 (20.5)	23 (32.9)	16 (13.3)	0.001
N2, n (%)	34 (17.9)	20 (28.6)	14 (11.7)	0.003

Data are reported as numbers (%) or means ± SD as appropriate. Abbreviations: ER = estrogen receptor; HER2 = human epidermal growth factor receptor 2; PR = progesterone receptor; IDC = invasive ductal carcinoma.

**Table 2:** Oncology management stratified by concomitant use of adjuvant goserelin and tamoxifen.

Characteristics	Overall Population n = 190	Goserelin and Tamoxifen n = 70	No Goserelin n = 120	*p* Value
Neoadjuvant anthracycline chemotherapy, n (%)	72 (37.9)	43 (61.4)	29 (24.2)	<0.001
Neoadjuvant anthracycline dosages:				0.54
200 mg/m^2^, n (%)	1 (1.4)	1 (2.3)	0 (0)	
210 mg/m^2^, n (%)	1 (1.4)	1 (2.3)	0 (0)	
220 mg/m^2^, n (%)	5 (6.9)	2 (4.7)	3 (10.3)	
240 mg/m^2^, n (%)	65 (90.3)	39 (90.7)	26 (89.7)	
Mean dose, mg/m^2^	237.6 ± 7.6	237.4 ± 8.5	237.9 ± 6.2	0.79
Neoadjuvant paclitaxel chemotherapy, n (%) *	37 (19.5)	20 (28.6)	17 (14.2)	0.01
Neoadjuvant trastuzumab chemotherapy, n (%) **	37 (19.5)	20 (28.6)	17 (14.29	0.01
Quadrantectomy, n (%) ***	98 (51.6)	40 (57.1)	58 (48.3)	0.24
Adjuvant anthracycline chemotherapy, n (%)	99 (52.1)	14 (20.0)	85 (70.8)	<0.001
Adjuvant anthracycline dosages:				0.31
200 mg/m^2^, n (%)	1 (1.0)	0 (0)	1 (1.2)	
210 mg/m^2^, n (%)	2 (2.0)	0 (0)	2 (2.4)	
220 mg/m^2^, n (%)	9 (9.1)	0 (0)	9 (10.6)	
230 mg/m^2^, n (%)	5 (5.1)	2 (14.3)	3 (3.5)	
240 mg/m^2^, n (%)	82 (82.8)	12 (85.7)	70 (82.4)	
Mean dose, mg/m^2^	236.7 ± 8.1	238.6 ± 3.6	236.4 ± 8.6	0.34
Adjuvant trastuzumab chemotherapy, n (%)	57 (30.0)	20 (28.6)	37 (30.8)	0.74
Adjuvant paclitaxel chemotherapy, n (%)	118 (62.1)	27 (38.6)	91 (75.8)	<0.001
Adjuvant docetaxel chemotherapy, n (%)	16 (8.4)	10 (14.3)	6 (5.0)	0.02
Adjuvant tamoxifen therapy, n (%)	111 (58.4)	70 (100.0)	41 (34.2)	<0.001
Radiotherapy	97 (51.1)	40 (57.1)	57 (47.5)	0.20

Data are reported as numbers (%) or means ± SD as appropriate. * Neoadjuvant paclitaxel dosage 85 mg/m^2^. ** Neoadjuvant trastuzumab dosage 6 mg/kg. *** Quadrantectomy vs. mastectomy (all patients underwent breast surgery).

**Table 3:** Cardiac measurements and outcomes at baseline and during follow-up stratified by concomitant use of adjuvant goserelin and tamoxifen.

Cardiac Measurements	Overall Population n = 190	Goserelin and Tamoxifen n = 70	Controls No Goserelin n = 120	*p* Value
LVEF at baseline, %	65.8 ± 1.9	66.1 ± 2.2	65.7 ± 1.7	0.21
LVEF at 3-month, %	65.5 ± 2.3	65.7 ± 2.7	65.3 ± 2.1	0.27
LVEF at 6-month, %	65.3 ± 2.6	65.5 ± 2.9	65.1 ± 2.5	0.34
LVEF at 1-year, %	64.7 ± 3.2	65.0 ± 3.2	64.6 ± 3.1	0.29
Hear rate at 3-month, bpm	65.8 ± 8.3	66.2 ± 8.5	65.5 ± 8.2	0.54
Hear rate at 6-month, bpm	67.0 ± 8.5	64.3 ± 8.4	68.6 ± 8.1	<0.001
Hear rate at 1-year, bpm	66.9 ± 8.6	65.3 ± 8.5	67.8 ± 8.5	0.05
QRS at 3-month, ms	87.9 ± 5.9	86.9 ± 6.0	88.5 ± 5.8	0.06
QRS at 6-month, ms	87.4 ± 4.4	86.9 ± 4.4	87.7 ± 4.4	0.18
QRS at 1-year, ms	87.4 ± 4.2	87.6 ± 3.9	87.2 ± 4.3	0.38
QTc at 3-month, ms	379.4 ± 10.8	378.5 ± 11.0	380.0 ± 10.7	0.34
QTc at 6-month, ms	379.4 ± 10.9	379.8 ± 10.7	379.1 ± 11.0	0.66
QTc at 1-year, ms	378.9 ± 10.7	378.1 ± 10.6	379.4 ± 10.8	0.40
Outcomes				
LVEF difference at 1-year, %,	1.11 ± 2.8	1.03 ± 2.5	1.16 ± 2.9	0.73
LVEF reduction ≥ 10% at 1-year, n (%)	9 (4.7)	3 (4.3)	6 (5.0)	0.82
LVEF < 65% at 1-year, n (%)	29 (15.3)	10 (14.3)	19 (15.8)	0.77

Data are reported as numbers (%) or means ± SD as appropriate. Abbreviations: LVEF = left ventricular ejection fraction; QTc = corrected QT interval.

**Table 4:** Multivariate logistic regression models.

Treatment Strategy	LVEF < 65% at 12 Months		LVEF Reduction ≥ 10% at 12-Month	
	OR (95% CI)	*p* Value	OR (95% CI)	*p* Value
Model 1: age adjusted				
No hormone therapy	1 (reference)		1 (reference)	
Only tamoxifen	1.65 (0.56–4.90)	0.36	2.95 (0.46–18.62)	0.24
Goserelin and tamoxifen	0.95 (0.36–2.51)	0.92	0.90 (0.16–4.81)	0.90
Model 2: age and radiotherapy				
No hormone therapy	1 (reference)		1 (reference)	
Only tamoxifen	1.53 (0.5–4.71)	0.39	3.08 (0.47–19.93)	0.23
Goserelin and tamoxifen	0.99 (0.37–2.65)	0.99	0.88 (0.16–4.70)	0.88
Model 3: age, adjuvant chemotherapy and radiotherapy				
No hormone therapy	1 (reference)		1 (reference)	
Only tamoxifen	2.07 (0.37–11.46)	0.40	3.29 (0.18–57.13)	0.41
Goserelin and tamoxifen	1.24 (0.26–5.83)	0.77	0.88 (0.07–11.09)	0.92

Abbreviations: LVEF = left ventricular ejection fraction. Adjuvant chemotherapy agents: anthracyclines, trastuzumab; paclitaxel or docetaxel.

**Table 5:** Inverse probability of treatment weighting: clinical traits stratified by concomitant use of adjuvant goserelin and tamoxifen.

Characteristics	Goserelin and Tamoxifen n = 70	Controls No Goserelin n = 120	*p* Value
Age, mean ± SD, years	38.7 ± 4.4	38.9 ± 11.1	0.70
Cardiovascular risk factors			
Diabetes, %	5.1	3.5	0.81
Hypertension, %	6.3	6.7	0.95
Hypercholesterolemia, %	2.0	1.2	0.88
Current smoking, %	51.5	62.7	0.29
Obesity, %	25.6	21.0	0.64
Prior hormone therapy, %	10.2	8.9	0.87
Clinical features			
Side of disease (right), %	60.8	61.7	0.93
Ductal invasive carcinoma, %	70.0	81.0	0.11
HER2-positive, %	28.4	20.5	0.42
Stage T1, %	14.9	5.4	0.23
Stage T2, %	69.2	86.9	0.06
Stage T3, %	15.9	7.7	0.32
N0, %	44.8	59.7	0.10
N1, %	30.9	15.0	0.14
N2, %	24.3	8.3	0.06
Oncology management			
Neoadjuvant anthracycline, %	55.2	39.9	0.10
Neoadjuvant trastuzumab, %	28.4	12.2	0.08
Neoadjuvant paclitaxel, %	28.4	12.2	0.08
Mastectomy, %	40.1	32.2	0.45
Adjuvant anthracycline, %	29.9	55.1	0.06
Adjuvant trastuzumab, %	28.4	20.5	0.42
Adjuvant paclitaxel, %	44.9	59.0	0.10
Adjuvant docetaxel, %	11.7	3.8	0.28
Radiotherapy, %	59.9	67.4	0.47

Data are reported as % or means ± SD. Abbreviations: HER2 = human epidermal growth factor receptor 2.

## Data Availability

The original contributions presented in the study are included in the article, further inquiries can be directed to the corresponding authors.
